# Isolating structural errors in reaction networks in systems biology

**DOI:** 10.1093/bioinformatics/btaa720

**Published:** 2020-07-13

**Authors:** Woosub Shin, Joseph L Hellerstein

**Affiliations:** eScience Institute, University of Washington, Seattle, WA 98195-5061, USA; Department of Bioinformatics – BiGCaT, NUTRIM, Maastricht University, 6229 ER Maastricht, The Netherlands; eScience Institute, University of Washington, Seattle, WA 98195-5061, USA

## Abstract

**Motivation:**

The growing complexity of reaction-based models necessitates early detection and resolution of model errors. Considerable work has been done on the detection of mass balance errors, especially atomic mass analysis (AMA) (which compares the counts of atoms in the reactants and products) and Linear Programming analysis (which detects stoichiometric inconsistencies). This article extends model error checking to include: (i) certain structural errors in reaction networks and (ii) error isolation. First, we consider the balance of chemical structures (moieties) between reactants and products. This balance is expected in many biochemical reactions, but the imbalance of chemical structures cannot be detected if the analysis is done in units of atomic masses. Second, we improve on error isolation for stoichiometric inconsistencies by identifying a small number of reactions and/or species that cause the error. Doing so simplifies error remediation.

**Results:**

We propose two algorithms that address isolating structural errors in reaction networks. Moiety analysis finds imbalances of moieties using the same algorithm as AMA, but moiety analysis works in units of moieties instead of atomic masses. We argue for the value of checking moiety balance, and discuss two approaches to decomposing chemical species into moieties. Graphical Analysis of Mass Equivalence Sets (GAMES) provides isolation for stoichiometric inconsistencies by constructing explanations that relate errors in the structure of the reaction network to elements of the reaction network. We study the effectiveness of moiety analysis and GAMES on curated models in the BioModels repository. We have created open source codes for moiety analysis and GAMES.

**Availability and implementation:**

Our project is hosted at https://github.com/ModelEngineering/SBMLLint, which contains examples, documentation, source code files and build scripts used to create SBMLLint. Our source code is licensed under the MIT open source license.

**Supplementary information:**

Supplementary data are available at *Bioinformatics* online.

## 1 Introduction

Mathematical models are an essential part of science and engineering, because of their ability to explain complex phenomena and predict outcomes. In Systems Biology, many mathematical models are based on chemical reactions that specify how reactants are transformed into products. Herein, a reaction is specified by listing the chemical species (and their associated stoichiometry) for the reactants and the products. The accuracy of reaction-based models depends in large part on the correct specification of the reactions. Verifying reaction specifications has become quite challenging as reaction-based models have grown in complexity. For example, BioModels (Glont *et al.*, 2018; Malik-Sheriff *et al.*, 2020), a repository of literature-based physiologically and pharmaceutically relevant mechanistic models in standard formats, especially the Systems Biology Markup Language (SBML) (see Hucka, 2013), contains over 800 curated models that range in size from tens to thousands of reactions. There are many similar public repositories of biological models such as CellML (Lloyd *et al.*, 2008), MAMMOTh (Kazantsev *et al.*, 2018) and BiGG (King *et al.*, 2016; Norsigian *et al.*, 2019). The correctness of models in such public repositories is of particular concern since these repositories are often the starting point for new modeling projects.

How can we address the correctness of reaction-based models as they grow in complexity? Our answer draws inspiration from approaches used in software engineering (Hellerstein *et al.*, 2019). If we view reaction-based models as a kind of software, then the complexity of today’s reaction-based models is comparable to the complexity of computer software in the early 1960s when programs were typically tens to a few thousand statements. Today, open source software such as Linux and the Apache Web Server have several million statements. This thousandfold increase in complexity is in part due to the development of sophisticated tools that automate error checking of software codes. One example is ‘linters’ (e.g. Darwin, 1988) that check for errors such as a variable that is referenced before it is assigned and identify unreachable code (e.g. a statement that follow a return statement). Both of these are examples of *static* error checking that is done by examining source codes without requiring code execution (Louridas, 2006).

Our goal is to develop linters that facilitate the development of reaction-based models. A linter finds errors by analyzing specifications or statements without running simulation codes. One example of linting a reaction is detecting mass balance errors, discrepancies between the total mass of reactants and the total mass of products. Many have noted the importance of checking for mass balance errors (Brim *et al.*, 2013; Chaouiya *et al.*, 2013; Clark *et al.*, 2012; Medley *et al.*, 2016; Shaw *et al.*, 2012). One approach to detecting mass balance errors is atomic mass analysis (AMA). AMA uses annotations of chemical species to obtain atomic formulas (e.g. Misirli *et al.*, 2016; Neal *et al.*, 2019) and then looks for differences in the atoms in the reactants and products. Two examples of AMA implementation and associated tooling are the MEMOTE system (Lieven *et al.*, 2020) and the COBRA Toolbox (Heirendt *et al.*, 2019). Annotations used for AMA often specify atom ionization states, and so AMA can check both charge balance and mass balance.

The flexibility of AMA can be expanded by the use of ‘R groups’ to designate subparts of molecule (e.g. Brunk *et al.*, 2018). This is a convenient naming scheme that can be used even if the atomic formula of the R group is unknown. Although the formula may be unknown, the R group still represents a *single* atomic formula, and so a single R group cannot represent a chemical structure that has several atomic variants.

AMA provides great value by checking for the balance of individual atoms. But biochemical modelers often think in terms of chemical structures, a higher level of abstraction than individual atoms (e.g. Brunk *et al.*, 2018; Nelson and Cox, 2004). Indeed, the concept of chemically similar groups is at the heart of organic chemistry. Introductory texts typically discuss 30 or so such groups along with the reactions in which they participate (e.g. Wade, 2010). Further, the Gene Ontology is in part structured around molecular functions, many of which are about transferring chemical structures between molecules (e.g. Ashburner *et al.*, 2000). We use the term *moiety* to refer to a chemical structure; more specifically, a moiety is ‘a part or portion of a molecule, generally complex, having a characteristic chemical or pharmacological property’ (Brightman, 2003). Unlike an R group, a single moiety may refer to groupings of atoms that have slightly different atomic formulas.

Many reactions preserve the balance of moieties between reactants and products. Consider ATP hydrolysis. This reaction is commonly written as ATP → ADP + Pi. Pi is an inorganic phosphate moiety. ATP has one adenosine moiety and three inorganic phosphate moieties; ADP has one adenosine moiety and two inorganic phosphate moieties. The inorganic phosphate moieties in ATP, ADP and the unbound Pi have slightly different atomic formulas. For example, the *γ* phosphate in ATP has a shared oxygen atom, but the unbound inorganic phosphate does not. The reaction is moiety balanced because we have one adenosine and three phosphates in the reactants and the products. Occasionally, we see ATP hydrolysis written as ATP → ADP; clearly, this is not moiety balanced. Note that, using AMA with R groups is insufficient for detecting moiety balance errors since R groups refer to a single atomic formula, not chemical groups like inorganic phosphate whose atomic composition may vary.

Although the reaction ATP → ADP + Pi is moiety balanced, it is not mass balanced because of the differences in the atomic formulas of the inorganic phosphates. To obtain mass balance, we need to include water in the reactants. However, many modelers do not include water for reactions in solution because its concentration is large and relatively constant. Such molecules are often referred to as implicits. Another example of an implicit molecule is inorganic phosphate in the reaction ATP → ADP. Many modelers find it burdensome and even unnatural to include implicit molecules in their reaction networks. In these cases, checking mass balance may be less meaningful, and so such checks should be optional. This could be done in the same manner as with software linters, such as the disable flag (and in-line ‘disable comments’) in the pylint package. Finally, we note that even when mass balance checking is inappropriate, these reaction networks may well benefit from checking moiety balance in a way that handles implicit moieties.

The foregoing motivates our generalizing from mass (and charge) balance to include additional structural errors in reaction networks. We note that others have considered structural errors such as blocked reactions and unreachable reactions (e.g. Lieven *et al.*, 2020). Herein, we consider *moiety balance errors*. A moiety balance error is present if a reaction that should preserve moiety balance is incorrectly specified so that the count of moieties in the reactants differs from its count in the products. Moiety preserving reactions are exceedingly common in biochemistry. For example, there are a large number of reactions with transferases that facilitate the transfer of a chemical group from a reactant to a product. That said, not all reactions preserve moiety balance, and so there is a need for selective disabling of checking for moiety balance similar to the need for selective disabling of checking for mass balance. Further, some moieties may be implicit (as with inorganic phosphate in the reaction ATP → ADP), and so it is also desirable to optionally ignore moiety balance for some moieties.

**Fig. 1.**
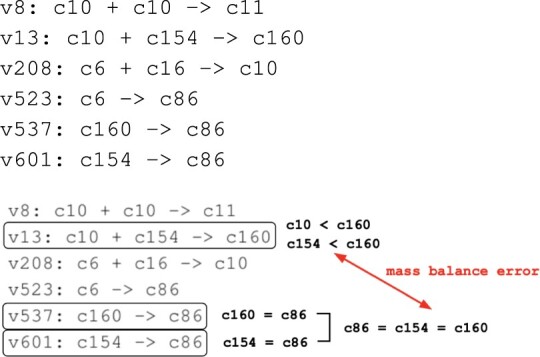
Snippet of BIOMD0000000255 that contains a stoichiometric inconsistency. Reactions are identified by the label that precedes the colon. A stoichiometric inconsistency is caused by the three reactions in boxes (v13, v537, v607) that constrain the relative masses of the four chemical species (c10, c86, c154, c160) in bold

Our notion of a structural error also includes errors in the structure of the reaction network. In particular, we consider *stoichiometric inconsistency*, a structural error that implies that one or more chemical species have a mass of zero. We illustrate this with an example from BioModels. Consider the model BIOMD0000000255, a model with 827 reactions.Figure 1 displays 6 of the model’s 827 reactions expressed as chemical equations. We analyze this model as follows. Reactions v537 and v601 imply mass equality between chemical species because these reactions have a single reactant and a single product, and all stoichiometries are one. Similarly, reaction v13 implies that the mass of c160 is larger than the mass of either c10 or c154 (since all chemical species must have non-zero mass). Thus, we conclude a contradiction, that the mass of c160 must be larger than its own mass.

**Fig. 2.**
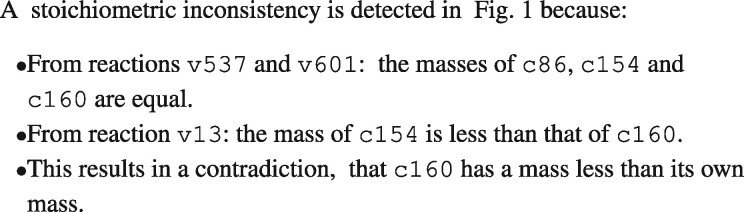
Explanation for the stoichiometric inconsistency in Figure 1. The narrative shows how the RIS v13, v537 and v607 and the SIS c10, c86, c154 and c160 cause in a stoichiometric inconsistency

Much of our focus is on error isolation, finding a subset of the chemical species and reactions that explain an error. Figure 2 displays an explanation for the foregoing structural error based on the relationships implied by the reaction network. The explanation involves a subset of reactions and chemical species that are implicated in the error. We use the terms reaction isolation set (RIS) and species isolation set (SIS) to refer to these reactions and chemical species.

Generalizing the example in Figure 2, we define *error isolation* as comprising: (i) an RIS, (ii) an SIS and (iii) a computationally simple explanation that shows how the RIS and SIS cause the error. By computationally simple, we mean that a human can readily understand how the RIS and SIS cause the error. There are two reasons for requiring that the explanation be computationally simple. First, a human should be able to verify the detection by inspection since complex detection algorithms may have software bugs and/or numerical problems. Second, errors can be remediated in many ways, and so it is important to expose causal relationships so that the options for remediation are clear. For example, two ways to remediate the error in Figure 1 are: (i) add c10 to the products of reaction v537; and (ii) delete c10 from the reactants of v13. In general, we want both the RIS and SIS to be small. Indeed, if one is small, the other tends to be small as well. We mostly use RIS in our studies since reactions relate more directly to the structure of the reaction network.

Little work has been done on error isolation in reaction networks. The absence of error isolation seems to pose little problem for AMA as it is used in current practice. This is because AMA is used mostly for metabolic models, as in MEMOTE. Since the reaction isolation set for AMA is always one, isolation is about the SIS (chemical species). For metabolic models, these are small molecules, and so isolation is less problematic. However, models of signaling systems and regulated pathways involve proteins with thousands of atoms that make the SIS more complex and hence complicate error isolation for AMA.

For stoichiometric inconsistencies, error isolation involves both the RIS and SIS. Recall that a stoichiometric inconsistency is present if there is *no* assignment of positive masses to chemical species such that all reactions are mass balanced. More formally, consider the stoichiometry matrix *N*, a matrix whose *ij*th entry is the moles of a chemical species *i* which is produced by reaction *j*. (This entry is negative if the reaction consumes species *i*.) A stoichiometric inconsistency is present if there is no vector of positive masses $v=\{v_{i}\}$ such that $N^{T}v=0$.

In current art, stoichiometric inconsistency is detected by formulating a linear programming (LP) feasibility problem with the constraints $N^{T}v=0$ and $v_{i}>0$ for each *i* (Gevorgyan *et al.*, 2008; Nikolaev *et al.*, 2005). We refer to this as *LP analysis*. Several tools implement this approach (e.g. Heirendt *et al.*, 2019; Lund Steffensen *et al.*, 2016; Swainston *et al.*; 2011). Although stoichiometric inconsistency relates to the structure of the reaction network, it also relates to mass balance: all stoichiometric inconsistencies are mass balance errors. However, not all mass balance errors are stoichiometric inconsistencies. For example, the model consisting of the single reaction ATP → ADP contains a mass balance error, but there is no stoichiometric inconsistency.

LP analysis is intended for error detection, not error isolation. However, there are extensions to LP analysis that use multiple steps of Mixed Integer Linear Programming (MILP) for error isolation (Gevorgyan *et al.*, 2008; Orman *et al.*, 2011); we refer this MILP extended LP analysis as *xLP*. xLP calculates the equivalents of the SIS and RIS using three MILP steps; the last step is an iterative optimization over the results of the second step. Supplementary Section S7 details several issues with xLP: (i) it does not provide an explanation that relates stoichiometric inconsistencies to the SIS and RIS; (ii) we know of no system that implements xLP, although there are implementations that simplify or eliminate one or more steps such as MEMOTE (Lieven *et al.*, 2020) and the COBRA Toolbox (Heirendt *et al.*, 2019); and (iii) xLP scales very poorly, as evidenced by our analysis of xLP on Recon3D in Supplementary Section S7.

This article addresses the isolation of structural errors in reaction networks. We propose *moiety analysis* for reactions that preserve moiety balance. As with AMA, moiety analysis verifies that there are equal counts in the reactants and products. AMA works in units of atoms; moiety analysis works in units of moieties. As argued previously, it is valuable to analyze moiety balance separate from mass balance. Further, working in units of moieties can have the additional benefit of identifying imbalances at a higher level of abstraction (see Supplementary Section S1). As with the annotation standards that support AMA, broad use of moiety analysis will require standards as well. We have developed tooling to aid in obtaining the moiety structure of molecules so that modelers can evaluate moiety analysis in support of future standards efforts. We emphasize that our contribution here is motivating, defining and providing practical illustrations of moiety analysis. Although we propose two schemes for representing moiety structures as a way to demonstrate the value of moiety analysis, we do not claim any contribution related to representing molecular structures. Examples of such representations include: PDB files (Berman *et al.*, 2003), bigSMILES (Lin *et al.*, 2019), HELM (Zhang *et al.*, 2012) and BpForms (Lang *et al.*, 2020).

We also address the isolation of stoichiometric inconsistencies in reaction networks. We propose Graphical Analysis of Mass Equivalence Sets (GAMES). As with LP analysis, GAMES detects stoichiometric inconsistencies. GAMES improves on the error isolation capabilities of xLP by using a combination of graphical analysis and linear algebra to explain errors in terms of their RIS and SIS, such as the explanation in Figure 2.

We have created open source, pip distributable implementations of moiety analysis and GAMES along with a tool for detection using LP analysis.

## 2 Materials and methods

This section describes approaches we propose for isolating structural errors in reaction networks.

### 2.1 Moiety analysis

Moiety analysis is an extension of AMA that uses moieties instead of atoms as the units of comparison. Indeed, balance checking can be generalized to work with arbitrary units, as illustrated in Supplementary Section S1 that describes a generalized balance checking algorithm.

There are many examples of moieties. The three phosphate groups in ATP (along with the inorganic phosphate molecule) have slightly different atomic formulas, but they are all instances of the same Pi moiety. Other examples of moieties include acetyl, methyl and amine chemical groups. Moieties differ from R groups in that a single moiety can refer to many atomic structures. Moieties differ from stochastic representations (e.g. bigSMILES) in that moieties are deterministic; that is, a molecular structure either is or is-not an instance of a moiety. Having deterministic specifications of chemical structures is essential for deterministic checking of balance errors.

Moiety analysis checks each moiety to determine that its occurrence count in the reactants equals its occurrence count in the products. To illustrate, consider ATP → ADP + Pi with the moieties A and Pi; the latter also refers to the bound moieties in ATP and ADP. The occurrence count of A is one in both the reactants and products, and the occurrence count of Pi is three in both reactants and products. So, there is moiety balance. However, as noted before, this reaction is not mass balanced.

Although moiety balance does not guarantee mass balance, it can sometimes be used in combination with mass balance to simplify error isolation. Consider the mitogen activated protein kinase (MAPK) cascade in BIOMD0000000011 (Levchenko *et al.*, 2000). MAPK is a large molecule that has ∼360 amino acids, about 3000 individual atoms. Consider an *incorrectly* written version of Reaction 19 in the model: Reaction19a: MAPK + MEKpp → MEKpp, where MEKpp is a doubly phosphorylated extracellular regulated kinase. Clearly, we are missing a MAPK in the products. Instead of reporting this, an analysis in units of atoms would report that the reactants have an excess of ∼3000 atoms of carbon, oxygen, nitrogen and sulfur. Although the large number of atoms clearly indicates that one or more large molecules are involved, it may not be obvious which large and/or small molecules contribute to the imbalance, thereby complicating error remediation.

Now consider analyzing the above reaction using moiety analysis. The moieties are MAPK, MEK and p. For MEK and p, the count of moieties is the same for reactants and products. However, there is one MAPK in the reactants, and none in the products. So, the modeler looks at the reactants for an extra MAPK and at the products for a missing MAPK. This seems much more useful than an accounting of 3000 missing atoms. Supplementary Section S1 illustrates an algorithm that integrates AMA with a kind of moiety analysis to improve the isolation of mass balance errors.

The main barrier to using moiety analysis is annotating molecules with their moiety structure. There are two parts to overcoming this barrier: (i) addressing the feasibility of decomposing molecules into moieties and (ii) providing standards and tools to accomplish the annotations. Regarding feasibility, we note that choosing an appropriate representation of molecules is a kind of modeling that depends on the chemical network. We do not claim that moieties are always the best representation. That said, there is ample evidence that decomposition into molecular substructures works well for some chemical networks as evidenced by: (i) the use of R groups in biochemical reactions; (ii) the hierarchical representations of molecules used in rule-based systems (e.g. Chylek *et al.*, 2015) and HELM representations and (iii) examples we provide in the Supplementary Information in which chemical species are decomposed into moiety structures for several models in BioModels.

The second challenge for moiety analysis is providing standards and tools. We have previously mentioned a rich set of existing techniques for representing molecules: PDB files, bigSMILES, HELM, BpForms and rule-based representations. The choice of representation of moieties is a significant endeavour in its own right that is beyond the scope of this article. Instead, we propose a couple of simple representations and supporting tools that allow the modeling community to evaluate the value of moiety analysis before undertaking efforts to choose the best representation of moiety structures.

The first representation of moiety structures used in this article is an implicit annotation of molecules with their moiety structure using a naming convention for chemical species that is compatible with the SBML community standard (Hucka, 2013). We propose the following naming conventions to accomplish implicit annotations of moiety structures:

- *Naming Convention NC-1*: The name of a chemical species (in SBML, its id attribute) should be the concatenation of the names of the constituent moieties of the species (with repetitions for multiple occurrences of moieties); and
- *Naming Convention NC-2*: An underscore (‘_’) separates moiety names in the name of the chemical species.

Using this convention, ATP is written as A_Pi_Pi_Pi. There are several examples of this convention in BioModels (e.g. BIOMD0000000009). Supplementary Section S2 discusses some refinements of this convention that are supported by our implementation of moiety analysis.

Also discussed in Supplementary Section S2 is a way to explicitly represent the moiety structure of a chemical species. Constructing explicit representations can be cumbersome, and so we provide tooling to assist. Specifically, we noticed that many models in BioModels satisfy NC-1 but not NC-2. An example is BIOMD0000000011 referenced above in which MEKpp has one instance of the moiety MEK and two instances of the moiety p. Thus, in Supplementary Section S2, we describe the tool make_moiety_structure that takes as input: (i) an SBML file with species whose id attributes comply with NC-1 and (ii) a list of moieties. The tool outputs the moiety structure of the molecules in the SBML file.

### 2.2 Graphical Analysis of Mass Equivalence Sets

Stoichiometric inconsistency is a structural error in the reaction network that involves mass balance. A stoichiometric inconsistency implies that one or more chemical species must have a mass of zero. The most widely used approach for detecting stoichiometric inconsistencies is LP analysis. However, as noted previously, LP analysis is *not* intended for error isolation. Further, xLP, the MILP extensions to LP analysis for error isolation, have serious shortcomings, as detailed in Supplementary Section S7.

Here, we introduce the GAMES algorithm for detecting stoichiometric inconsistencies. GAMES provides error isolation by explaining errors in terms of their SIS and RIS. The explanations are constructed by inferring relationships between the masses of chemical species based on reactions in the model. Because the focus of GAMES is mass balance, we use the term mass balance error in the discussion of GAMES.

The GAMES algorithm consists of two parts. Section 2.2.1 describes the core algorithm, which we call basic GAMES or bGAMES. bGAMES uses graphical techniques to identify the SIS and RIS and then constructs an inference chain that relates the stoichiometric inconsistency to the SIS and RIS. Section 2.2.2 describes extensions to this algorithm that use linear algebra to construct pseudo reactions that infer the stoichiometric inconsistency from the RIS and SIS.

#### 2.2.1 Isolating errors with basic GAMES

Basic GAMES (bGAMES) can be viewed as a kind of ‘inference engine’ that attempts to construct inferences that contradict the assumption that all chemical species have positive mass. The inference steps rely on mass equality and inequality relationships implied by model reactions. If bGAMES infers that a chemical species does not have a positive mass, then the RIS and SIS are the set of the reactions and chemical species (respectively) that are used in the inference.bGAMES uses insights such as those used in Figure 1 to construct equality and inequality relationships between the masses of chemical species. For example in Figure 1, v537: c160 → c86 implies that the mass of c160 must be the same as the mass of c86. This is an example of a *uni–uni* reaction, a reaction with one reactant and one product that have the same stoichiometries. A uni–uni reaction implies that the mass of the reactant equals the mass of the product.

The equality relationships implied by uni–uni reactions allow bGAMES to construct groupings of chemical species that have the same mass. We refer to such a grouping as a Mass Equivalence Set (MEQ). From the previous example discussed in Figure 1, we see that reaction v537 infers a MEQ that consists of the species c160 and c86, which we denote by {c86 = c160}. MEQs are expanded by transitivity. To illustrate, Figure 1 also contains the MEQ {c154 = c86} (implied by v601), and so by transitivity, we have {c86 = c154 = c160}. To account for all species in Figure 1, we also have three singleton MEQs: {c10}, {c16} and {c11}.bGAMES also infers mass inequalities. For example, two mass inequalities can be inferred from the reaction v13: c10 + c154 → c160: (i) the mass of c160 is greater than the mass of c10 and (ii) c160 is greater than c154. v13 is an example of a *multi–uni* reaction, in which the reactants (or products) consist of two or more chemical species and there is a single chemical species as the product (or reactant).

The bGAMES inference engine uses uni–uni and multi–uni reactions to construct a directed graph that is then analyzed to detect stoichiometric inconsistencies. We refer to this as the *MEQGraph* since the nodes of the graph are MEQs. If (*X*, *Y*) is an arc in the MEQGraph, then the mass of MEQ *X* is less than the mass of MEQ *Y*. MEQs are constructed from the transitive closure of uni–uni reactions, and arcs are constructed from multi–uni reactions. For example, consider the reaction a + b → c, with a in MEQ *X*, b in MEQ *Y* and c in MEQ *Z*. Then, the MEQGraph has the arcs (*X*, *Z*) and (*Y*, *Z*).bGAMES detects a stoichiometric inconsistency by finding a cycle in the MEQGraph. This is because a cycle implies a logical contradiction, that all MEQs in the cycle have a mass less than their own mass.

Having detected a cycle in the MEQGraph, the RIS consists of reactions that are either: (i) multi–uni reactions that correspond to an arc in the cycle or (ii) uni–uni reactions used in the MEQs traversed by the cycle. The SIS are the chemical species associated with each MEQ. For example, in Figure 1 (with more details in Supplementary Fig. S3), there is a cycle with arcs labelled with reactions v13 and v208. The MEQs in this cycle are {c10} and {c6 = c86 = c154 = c160}. The latter are created by three uni–uni reactions (v523, v537, v601). The resulting RIS is {v13, v208, v523, v537, v601}; the SIS is {c6, c10, c86, c154, c160}.

The foregoing allows for constructing a narrative such as that shown in Figure 2. The narrative consists of three parts: (i) mass equivalences (MEQs) inferred from uni–uni reactions, (ii) mass inequalities obtained from multi–uni reactions and (iii) a statement of the contradiction inferred.bGAMES does not detect all stoichiometric inconsistencies. Since we can use LP analysis to *detect* stoichiometric inconsistencies, there is no false negative. However, since bGAMES can only explain stoichiometric inconsistencies that it detects, there is limited coverage for error isolation. By *coverage*, we mean the number of models in which bGAMES detects a stoichiometric inconsistency divided by the number of models in which LP analysis detects a stoichiometric inconsistency. The coverage limitations of bGAMES are largely due to its not analyzing multi–multi reactions, reactions with more than one chemical species for both reactants and products. In BioModels, bGAMES has a coverage of about 78%.

#### 2.2.2 Isolating errors with extended GAMES

This section describes extended GAMES (xGAMES), an extension to bGAMES that addresses the coverage limitations of bGAMES. SBMLLint automatically invokes xGAMES if bGAMES does not detect a stoichiometric inconsistency.

**Fig. 3.**
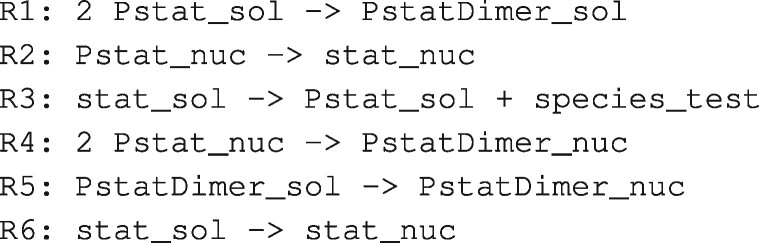
Reactions in BIOMD0000000167. The reaction names are changed to simplify the description. The complete model has two instances of R6 with different kinetics

We illustrate the capabilities of xGAMES using BioModels BIOMD0000000167 as displayed in Figure 3. Consider the following two reactions: R2: Pstat_nuc → stat_nuc and R4: 2 Pstat_nuc → PstatDimer_nuc. If these reactions are mass balanced, then the sum of the reactants of both reactions must have the same mass as the sum of their products. That is, the mass of 3 Pstat_nuc must equal the sum of the masses of stat_nuc and PstatDimer_nuc. Put differently, we have mass balance for the hypothetical reaction R2+R4: 3 Pstat_nuc → stat_nuc + PstatDimer_nuc. We do not claim that this reaction is chemically feasible, and so refer to it as a *pseudo reaction*. In this case, mass balance holds, and so we use the term *mass-balanced pseudo reaction*.

We can generalized beyond the summation of two reactions. If a collection of reactions $\left\{R_{i}\right\}$ is mass balanced, then any linear combination of these reactions will be a mass-balanced pseudo reaction.

LP analysis detects a stoichiometric inconsistency if there is no positive vector of masses *v* such that $N^{T}v=0$, where *N* is the stoichiometry matrix (columns are reactions and rows are species). Such a *v* cannot be found if either: (i) the dimension of the column (species) null space of *N^T^* is 0; or (ii) the column null space does not intersect the subspace where *v* is positive. xGAMES uses matrix decomposition to test for these conditions and do error isolation by reporting the reactions that result in a stoichiometric inconsistency.x. GAMES works with a new stoichiometry matrix (*N*) whose rows are MEQs instead of chemical species. Doing so allows us to eliminate uni–uni reactions since this information is already encoded in the MEQs. This is illustrated in Table 1. The columns are the pseduoreactions PR3, PR1 and PR4 that correspond to the original reactions R3, R1 and R4. For example PR1: 2{Pstat_sol} → {PstatDimer_sol=PsatDimer_nuc}. Note that, every column contains at least one negative value and at least one positive value. This is because every reaction has at least one reactant and at least one product.

**Table 1.** Stoichiometry matrix for pseudo reactions

MEQ	PR3	PR1	PR4
{species_test}	1	0	0
{Pstat_sol}	1	−2	0
{PstatDimer_sol=PstatDimer_nuc}	0	1	1
{Pstat_nuc=stat_nuc=stat_sol}	−1	0	−2

*Note*: Rows are MEQs. Columns are pseudo reactions that are numbered corresponding to the reactions in Figure 3. Cells are the stoichiometry of a MEQ in the products minus the stoichiometry of the MEQ in the reactions. Uni–uni reactions are not included since they are used to construct the MEQs.

Next, xGAMES transforms *N* into reduced column echelon form using standard techniques from linear algebra such as LU decomposition (Horn and Johnson, 1985). We denote this transformed matrix by $N^{R}=\{n_{ij}^{R}\}$. Table 2 displays *N^R^* for our running example. We can see that the leading entry in each column is non-zero and $n_{ij}^{R}=0$ for *j *>* i*, as required by reduced column echelon form. Also, as with Table 1, the columns of Table 2 are pseduo reactions. Specifically, the columns of *N^R^* are linear combinations of the columns of *N*. For example, $PR3^{\prime }=PR3+\frac{1}{2}PR1-\frac{1}{2}PR4$.x GAMES detects a mass balance error using the following decision criteria:

**Table 2.** Reduced column echelon form for the running example

MEQ	PR3′	PR1′	PR4
{species_test}	1	0	0
{Pstat_sol}	0	1	0
{PstatDimer_sol=PstatDimer_nuc}	0	0	1
{Pstat_nuc=stat_nuc=stat_sol}	0	−1	−2

*Note*: The prime symbol indicates a reaction formed by linear combinations of these in Table 1.

- **Detection Criteria (DC)**: A mass balance error is present if there is a linear combination of columns of *N* whose non-zero values all have the same sign.

It turns out that DC is a sufficient but not a necessary condition for stoichiometric inconsistencies, as we discuss later. Also, we note in passing that DC relates to the concept of a leakage mode in Gevorgyan *et al.* (2008), which is a set of reactions that results in inconsistent stoichiometry.

DC detects mass balance errors by identifying reactions for which mass is either created or destroyed. A column of the stoichiometry matrix in which all non-zero values are positive describes a reaction that has products and no reactants. That is, mass is created. Analogously, a column in which all non-zero values are negative describes a reaction that has reactants and no products, and so mass is destroyed.xGAMES detects a stoichiometric inconsistency by inferring that DC holds using proof by contradiction. The inference engine starts by assuming that the original reactions are mass balanced. From the foregoing, we know that linear combinations of mass-balanced reactions result in a mass-balanced pseudo reactions. So, if some linear combination of reactions in the model results in DC being satisfied, then we know that the original set of reactions is *not* mass balanced.

**Fig. 4.**
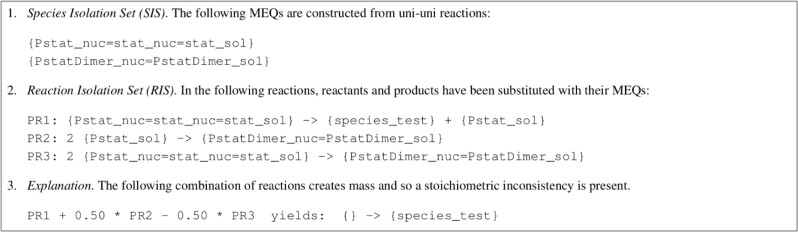
Example of xGAMES explanation for BIOMD0000000167. The SIS is a collection of MEQs; the RIS is a collection of pseudo reactions; the explanation is a ‘proof by contradiction’ that shows a linear combination of the RIS results in mass creation

An xGAMES explanation extends the analysis of bGAMES by considering pseudo reactions. This is illustrated in Figure 4 for BIOMD0000000167 (with complete details in Supplementary Fig. S6).

Step 1 in Figure 4 is generated by reporting the uni–uni reactions used to construct MEQs (as described in Section 2.2.1). From this, a new stoichiometry matrix is constructed. This matrix: (i) replaces each chemical species with its corresponding MEQ, (ii) sums rows that correspond to the same MEQ and (iii) deletes columns corresponding to uni–uni reactions (since the information in uni–uni reactions is implied by using MEQs instead of chemical species).

Now we consider how xGAMES infers a stoichiometric inconsistency. Let *N* be the resulting stoichiometry matrix. We use LU decomposition (Horn and Johnson, 1985) to factor *N^T^* into *PLU*, where *P* is a permutation matrix and *L*, *U* are lower and upper triangular matrices, respectively. Since *L* is invertible, we can calculate $U=L^{-1}P^{-1}N^{T}$, and $U^{T}=NP{\left(L^{-1}\right)}^{T}$. The columns of $P{\left(L^{-1}\right)}^{T}$ reveal the linear combinations of reactions of *N* that result in the columns echelon form.

For some models, there are multiple possible permutation matrices *P*, and thus multiple possible *L*, *U* matrices. Different choices for these matrices can, in some cases, result in detecting different errors. Thus, it is sometimes helpful to run GAMES multiple times to gain a more complete picture of the mass balance errors present in a model.

Once the column echelon form is obtained, we can readily extend it to reduced column echelon form by elementary matrix operations so that we have a matrix that informs us of the linear combinations of columns of *N* that are used to construct the reduced column echelon matrix *R*. So, the RIS in Step 2 of the explanation is the set of reactions that correspond to the columns in this linear combination that have non-zero multipliers; the SIS in Step 1 are the species (rows) in which the RIS columns have non-zero values. The explanation in Step 3 is the linear combination of the RIS that results in mass creation or destruction.

In our experience with BioModels, xGAMES has a coverage of about 98.1%. To understand the 1.9% coverage gap, recall that a stoichiometric inconsistency is present if (1) there is no vector of masses *v* such that $N^{T}v=0$ (where *N* is the stoichiometry matrix) such that (2) *v *>* *0. LP analysis checks precisely for these two conditions. The xGAMES decision criteria DC detects violations of condition (1) if *N^T^* has a trivial null space. However, DC only approximates detection of violations to condition (2). Specifically, DC looks for columns where the non-zero values have the same sign. This is a sufficient but not a necessary condition for detecting violations of *v *>* *0.

## 3 Results

This section studies moiety analysis and GAMES in the context of the curated SBML models in the BioModels repository.

### 3.1 Studies of moiety analysis

**Fig. 5.**
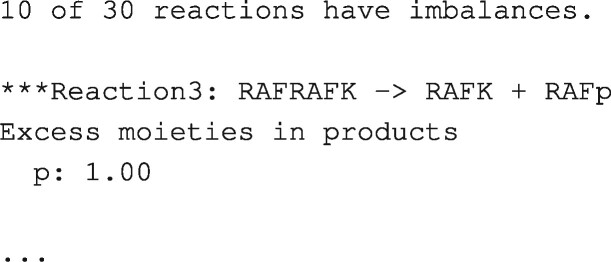
Partial moiety analysis report. This is the initial segment of the report generated by moiety analysis for BIOMD0000000011 using the moiety names RAF, K, p, PH, MEK and MAP. It appears that p (inorganic phosphate) is an implicit moiety that causes the mass imbalances

Moiety analysis reports and explains moiety imbalances. As shown in Figure 5, part of a report for BioModels BIOMD0000000011, the top of the report summarizes the errors detected; this is followed by a section for each reaction in which an imbalance is detected.

A core challenge with moiety analysis is exposing the moiety structure of chemical species. Our experience with BioModels has been that a non-trivial fraction of the curated models use names of chemical species that comply with our NC-1 naming convention. Consider BIOMD0000000167. The model has the species names Pstat_sol, PstatDimer_sol, stat_sol, Pstat_nuc, PstatDimer_nuc. These names are structured as follows: (i) P or a null string followed by (ii) stat followed by (iii) Dimer or null string followed by (iv) _sol or _nuc. We consider P, stat, Dimer, sol and nuc as candidate moieties for the model. With further scrutiny, we see that sol and nuc are not moieties; rather, this is how the modeler indicates compartments. We eliminate sol and nuc from our analysis by using the SBMLLint option to have a ‘configuration file’ that ignores these elements. We expose the moiety structure of the chemical species using the explicit representation of moiety structure as described in Section 2.1. This structure can be created automatically using the tool make_moiety_analysis.

Supplementary Section S2 contains more details of the algorithm used by make_moiety_structure. Supplementary Section S4 provides two in-depth case studies of applying moiety analysis to models in BioModels, and points to ∼10 examples of moiety analysis in our github repository.

### 3.2 Studies of GAMES

This section provides insights into the coverage and quality of explanations generated by GAMES. Supplementary Section S5 details two case studies of GAMES analysis done on models in BioModels.

To provide broader insights into GAMES, Table 3 displays results from analyzing the 826 curated models in which we report measures of the bGAMES and xGAMES portions of GAMES. We see that xGAMES has much better coverage than bGAMES, but xGAMES has much longer runtimes since higher coverage requires longer processing times. Another reason for longer runtimes for xGAMES is that the errors that are *not* covered by bGAMES are generally much more complicated. Optimization of the xGAMES code will likely reduce its runtimes. That said, these optimizations may be unnecessary, at least for model complexities comparable to BioModels, since the average time per model is a few seconds. This should be acceptable for model tooling, especially since GAMES should be run only if LP analysis reports a stoichiometric inconsistency. Supplementary Section S5 contains more case studies of GAMES, including very large models from BiGG.

**Table 3.** Runtimes for error isolation by bGAMES and xGAMES for 158 curated models

Algorithm	Total sec	per-Model sec	Num. errors	Coverage
bGAMES	52.73	0.43	123	77.8%
xGAMES	491.3	3.11	155	98.1%

*Note*: LP analysis confirmed that the models have mass imbalance. The first two data columns report the time to run the algorithms for all models and per model, respectively. The longer runtimes for xGAMES is large part due to its increased coverage means it detects more complex errors. Num. errors is the number of models with stoichiometric inconsistencies.

Next, we study the effectiveness of the error isolation produced by GAMES. The concern here is that if the SIS and/or RIS is very large, then the error isolation is not very useful. For example, consider the error isolation in Figure 4. Having a large RIS would greatly complicate Step 2 and Step 3. Thus, we quantify complexity using the size of the RIS. A related measure is the normalized RIS, the ratio of the RIS to the number of reactions in the model.

Table 4 reports the results. We see that in BioModels the RIS size is modest, averaging 3.78 for xGAMES and 5.56 for bGAMES.

**Table 4.** Isolation effectiveness for bGAMES and xGAMES

Algorithm	RIS size	Normalized RIS size
bGAMES	5.56	17.0%
xGAMES	3.78	11.6%

*Note*: Effectiveness is quantified in terms of the size of the reaction isolation set (RIS) in Step 2 of the explanations.

## 4 Discussion

The growing complexity of reaction-based models makes it challenging to detect and remediate model errors. This article focuses on isolating structural errors in reaction networks.

We consider two kinds of structural errors: imbalances of moieties in reactions and stoichiometric inconsistency (an error in the structure of the reaction network). We also consider error isolation since it exposes causal relationships that aid in error remediation. By isolating an error we mean: (i) finding a subset of chemical species that cause the error, which we call the species isolation set (SIS); (ii) finding a subset of the reactions that cause the error, which we call the reaction isolation set (RIS) and (iii) constructing a computationally simple narrative that explains the error in terms of the SIS and RIS. Further, the computationally simple narrative allows modelers to check for false detections. False detections are a concern because techniques that do computationally intensive matrix operations can suffer from numerical issues (e.g. near singularities) because of the structure of the stoichiometry matrix.

We identify two areas of work related to ours. Atomic mass analysis (AMA) is widely used to detect structural errors in the form of imbalances in atomic masses. But AMA is not intended to detect imbalances in chemical structures such as moieties that may have variations in their atomic formulas. For stoichiometric inconsistency, extended LP (xLP) uses multiple steps of mixed integer linear programs to calculate equivalents to the SIS and RIS. But xLP has issues with computational and algorithmic complexity and an inability to explain errors detected in terms of their SIS and RIS.

The first structural error that we address is the balance of moieties between reactants and products. Many biochemical reactions preserve this balance (e.g. reactions catalyzed by transferases). Moiety analysis can be viewed as an extension of AMA to moieties. Indeed, these algorithmic similarities mean that moiety analysis can be integrated with AMA to improve AMA error isolation by reporting a few missing moieties instead of a large number of missing atoms. The central challenge with moiety analysis is exposing the moiety structure of molecules. We introduce two approaches to exposing moiety structure that allow the modeling community to evaluate moiety analysis as a step toward the development of relevant standards and tools.

Our alternative to xLP analysis is GAMES. GAMES uses graphical techniques and linear algebra to explain a stoichiometric inconsistency in terms of its SIS and RIS. GAMES typically provides concise explanations for errors in models with several hundred to a couple thousand reactions. However, the GAMES SIS and RIS can be large for very big models.

We have created open source, pip installable implementations of moiety analysis and GAMES.

We are pursuing a number of directions for future work. For moiety analysis, our focus is the representations of the moiety structure of molecules. For GAMES, we are investigating ways to reduce runtimes, increase the coverage of stoichiometric inconsistencies, and better address error isolation when the RIS and/or SIS are large (as is the case for many BiGG models). For example, there might be a way to incorporate mathematical programming with graphical approaches that reduces runtimes without reducing coverage. Further, we are exploring revisions to the xGAMES detection criteria (DC) to increase the coverage of xGAMES. More broadly, we are interested in providing modelers with insights into preferred practices or ‘model patterns’, which we might be able to infer from analyzing many models.

## Supplementary Material

btaa720_Supplementary_Data
