# Supplementary material for: Isolating structural errors in reaction networks in systems biology
Source: Bioinformatics. 2020 Jul 13;37(3):388–95. doi: 10.1093/bioinformatics/btaa720 (PMC8058775; doi:10.1093/bioinformatics/btaa720)
Supplement: btaa720_Supplementary_Data [file btaa720_supplementary_data.pdf]

Isolating Structural Errors in Reaction Networks in Systems Biology: *Supplemental Information*

Woosub Shin and Joseph L. Hellerstein

S1 Generalized Balance Analysis

As noted in the main manuscript, AMA and moiety analysis use the same underlying algorithm to detect imbalances in reactions. These analyses differ only in the units being used: counts of atoms for AMA and counts of moieties for moiety analysis. The similarities of the underlying algorithms make it possible to provide two generalizations to balance analysis of reactions.

The first is a generalized algorithm for detecting imbalances in mass, charge, and moieties. Fig. S1 details a generalized balance analysis algorithm. The arguments of the algorithm are the reaction being evaluated and the function `units`. `units` takes a chemical species as an argument; it returns a vector appropriate for the analysis being conducted. For example, for AMA, the vector is the count of type of atom (e.g., 6 `C`, 12 `H`, 6 `O`). For charge balance, it is a scalar–the net charge. For moiety analysis, it is a vector count of moieties.

A second generalization of balance analysis relates to improving the error isolation of AMA. The idea is to augment how mass imbalances are reported by providing a higher level of abstraction–moieties that might be missing that could explain the mass imbalance. An algorithm that demonstrates this idea is shown in Fig. S2. The algorithm looks for missing (or extra) moieties that explain a discrepancy in mass between reactants and products.

S2 Exposing the Moiety Structure of Chemical Species

A core challenge for moiety analysis is the availability of the moiety structure of chemical species. Ideally, moiety structures should be provided through standards and tools that support the standards. Although such support may be available in the future, it is not available at this time.

Since we know of no computer readable source of moiety structures of chemical species, we propose two approaches to obtaining this information. This is intended as an interim solution to facilitate the evaluation of moiety analysis by the modeling community with an eye towards standards efforts.

```
balance_checker(reaction, units):
  /* Compute reactant units */
  reactant_units = 0
  for species in reactants:
    reactant_units += units(species)
  /* Compute product units */
  product_units = 0
  for species in products:
    product_units += units(species)
  /* Detect imbalances */
  return reactant_units - product_units
```

Fig. S1: *A general algorithm for checking of characteristics of chemical species in the reactants and products.* `units` *is a function of a species that returns an array appropriate for the characteristic being evaluated (e.g., counts of atoms, charges, counts of moieties) The algorithm returns the difference between the units in the reactants and products. A vector of zeroes indicates that there is no imbalance.*

The first approach is a convention for the names of chemical species (technically, their SBML id attribute). The `moiety_analysis` tool automatically extracts moiety structures for models that comply with naming conventions NC-1 and NC-2 described in Section 2.1. NC-1 requires that the occurrence of a moiety is indicated by the presence of the moiety name in the id attribute of the chemical species; NC-2 demands that these occurrences are separated by an underscore. For example, `ATP` is written as `A_Pi_Pi_Pi`. Because it is common to have multiple instances of a moiety in a chemical species, the naming convention also supports repetition counts. In this extended naming convention, moiety names are separated by a double underscore, and a repetition count follows the moiety name. For example, `A__Pi_3`. When repetition counts are used in chemical species, then a double underscore must be used to separate moieties.

A second approach to exposing moiety structures is through an explicit representation using a YAML file format (*https://en.wikipedia.org/wiki/YAML*). This has the advantage of imposing no requirement on the names of chemical species. For example, the moiety structure of ATP is expressed as:

```
- ATP:
  - A, 1
  - P, 3
```

`moiety_analysis` can be invoked with a `-config` option that specifies a YAML configuration file that contains a `moiety_structure` section with elements structured as in the `ATP` example above. Details of these file formats are described in the `README.md` file in the `SBMLLint github` repository.

Although this explicit representation avoids imposing restrictions on the

```
ama_with_moiety_isolation(reaction):
  difference = balance_checker(reaction, mass_units)
  if difference != 0:
    for moiety in moieties of reactants and products:
      if difference approximates mass_units(moiety):
        report possible missing moiety
```

Fig. S2: *Explaining mass balance errors with moieties. The algorithm attempts to explain a mass imbalance in terms of a missing (or extra) moiety.* `mass_units` *is a function that returns the atomic mass vector for a molecule or moiety.*

names of chemical species, it imposes a different burden – constructing explicit representations. Our experience is that many models in BioModels comply with NC-1, but far fewer models comply with NC-2 as well. For example, the chemical species `RAFRAFK` contains the moieties `RAF` and `RAFK`, but there is no separator between the moiety names.

This led us to develop the tool `make_moiety_structure` for models that comply with NC-1. The tool automatically generates the `moiety_structure` section of the `SBMLLint` configuration file. The tool takes as input the SBML model file and a list of moieties; the tool outputs the `moiety_structure` section of the configuration file. The tool handles cases where one moiety name is a sub-string of another moiety name (by searching for moiety names in decreasing order of their length). We have found this tool extremely helpful, although it is common that some edits are required to the automatically generated `moiety_structure` section.

The `SBMLLint` github repository contains approximately 10 case studies in which `make_moiety_structure` is used to expose the moiety structures of chemical species for models in BioModels. These case studies can be found in the folder `examples /moiety _analysis`. There is a separate sub-folder for each case study, and the sub-folders have the same structure (as documented in the `README.md`). Consider folder `11` for `BIOMD0000000011`. The file `moieties.yml` lists six moieties: `RAF`, `K`, `P`, `PH`, `MEK`, and `MAP`. Using the SBML and moiety files, `make_moiety_structure` creates moiety structures for the 22 chemical species in `BIOMD0000000011.xml`, as listed in the `moiety_structure` section of `config.yml`. `result.yml` contains the result of running the command: `moiety_analysis BIOMD0000000011 -config config.yml`:

```
0 of 30 reactions have imbalances.
```

The fact that we are able to so easily construct the moiety structure of `BIOMD0000000011` suggests that this model was developed with moieties in mind. We have found this to be the case for a non-trivial fraction of models in BioModels, about 10% to 20%. This suggests that for some modelers, it will be natural to expose the moiety structure of chemical species.

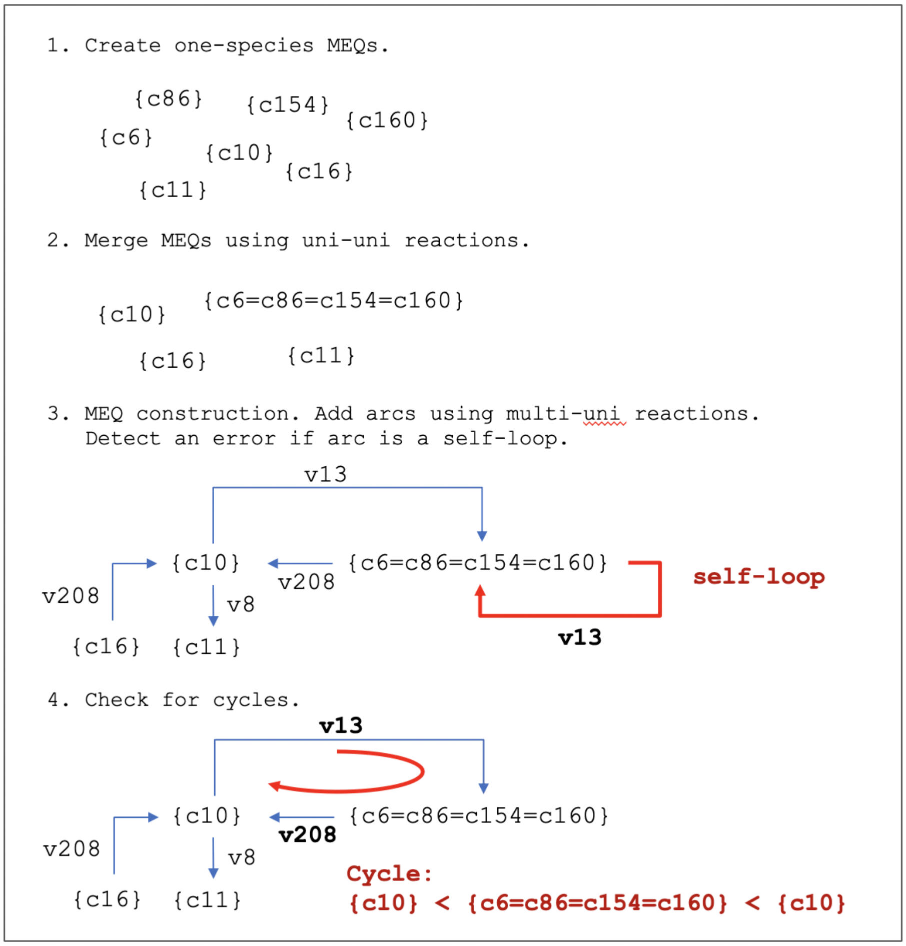

Fig. S3: Steps in the bGAMES algorithm. The figure illustrates the data structures used in each step of bGAMES for the reactions in Fig. 1. A mass balance error is detected if there is a loop in the graph since this implies a contradiction that some chemical species has a mass less than its own mass.

S3 Details of GAMES

Fig. S3 displays the steps in bGAMES for analyzing Fig. 1. bGAMES detects a stoichiometric inconsistency by finding a cycle in the MEQGraph. This is because a cycle implies a logical contradiction, that all MEQs in the cycle have a mass less than their own mass.

Below are described further details related to the bGAMES algorithm.

1. Initialization is done so that there is a MEQ for each chemical species.

2. MEQs are merged. This is done using uni-uni reactions to discover mass equivalences and detecting non-null intersections between sets. By merging MEQs A and B, we mean that a new MEQ C is formed such that $C = A \cup B$, and A, B are deleted.

3. Arcs are added between nodes (MEQs) based on multi-uni reactions to indicate strict inequality of masses.

4. The MEQGraph is checked for cycles. A cycle in the MEQGraph implies a logical contradiction, that some chemical species has a mass less than its own mass.

bGAMES addresses implicits by ignoring singleton implicit nodes and their incident arcs. For example in Fig. 1, if `c6` in reaction `v208` is an implicit, then we delete the arcs labelled `v208` and change the node `{c6=c160=c86=c154}` to `{c160=c86=c154}`. This leaves the self-cycle caused by `v13`, and so a mass balance error is still detected. Its RIS consists of `v13` and the uni-uni reactions that construct the MEQ `{c160=c86=c154}`.

Fig. S4 depicts the steps in xGAMES detection of mass balance errors. The algorithm stops when a mass balance error is detected. The algorithm does not attempt to find all stoichiometric inconsistencies for two reasons: (a) it is computationally expensive to report all mass balance errors; and (b) it is likely very confusing to the modeler to report all errors since one incorrect reaction specification may cause multiple mass balance errors.

There are a few caveats to the bGAMES and xGAMES algorithms. First, bGAMES only addresses stoichiometric inconsistencies caused by uni-uni and multi-uni reactions. Although xGAMES can also handle multi-multi reactions, there is some variability to the errors reported by xGAMES if there are multiple ways to decompose the stoichiometry matrix. As with LP analysis, xGAMES can be subject to numerical errors depending on the structure of the stoichiometry matrix. To the best of our knowledge, the only way to expand beyond stoichiometric inconsistencies is for the modeler to provide more information, such as is done with AMA and moiety analysis. Section S9 discusses potential improvements in the GAMES algorithms.

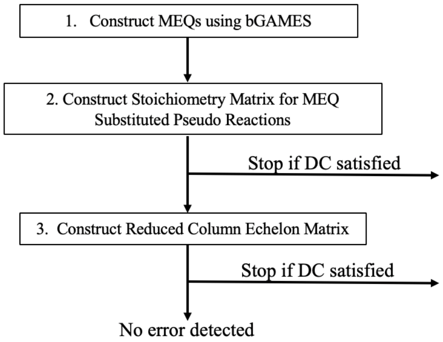

Fig. S4: Steps in the xGAMES algorithm. The algorithm stops when the first error is encountered using the decision criteria (DC).

S4 Additional Moiety Analysis Case Studies

We proceed with case studies of applying moiety analysis to two curated models in BioModels. Considered first is BIOMD0000000140 (Hoffmann *et al.* [2007]), a model of temporal control and selective gene activation in mammalian cells. The model has 45 reactions along with 65 parameters and chemical species. Many of the chemical species already use the NC-1, NC-2 naming convention for exposing moiety structures that we describe in Section 2.1. Even better, many of the reactions are moiety balanced. For example, `v1: NFkB + IkBalpha -> IkBalpha_NFkB`. This suggests that `NFkB` and `IkBalpha` should be treated as moieties. After examining other reactions, we selected the following as candidate moieties: `nuc, transcript, IKK, IkBbeta, IkBalpha, IkBeps, NFkB`. It appears that `nuc` is used to designate a compartment, and `transcript` to indicate a gene product. Thus, both of these are ignored in our analysis by making use of the `ignored_moieties` section of the configuration file. Fig. S5 displays the results of doing moiety analysis under these conditions. We see that `IkBalpha, IkBbeta, IkBeps` are often missing in reactions. Very likely this is because of implied proteolysis in the model. If we wanted to change the model so that proteolysis is explicit, we can introduce a `proteolysis` moiety that appears in the reactants. For example `v5` can be re-written as `IKK_IkBalpha_NFkB` $\rightarrow$ `NFkB + IkBalpha_proteoloysis + IKK`, and then we would add `proteolysis` to the `ignored_moieties` section of the configuration file.

```
9 of 45 reactions have imbalances.

***v5: IKK_IkBalpha_NFkB -> NFkB + IKK
Excess moieties in reactants
  IkBalpha: 1.00

***v7: IKK_IkBbeta_NFkB -> NFkB + IKK
Excess moieties in reactants
  IkBbeta: 1.00

***v9: IKK_IkBeps_NFkB -> NFkB + IKK
Excess moieties in reactants
  IkBeps: 1.00

***v10: IkBalpha_NFkB -> NFkB
Excess moieties in reactants
  IkBalpha: 1.00

***v11: IkBbeta_NFkB -> NFkB
Excess moieties in reactants
  IkBbeta: 1.00

***v12: IkBeps_NFkB -> NFkB
Excess moieties in reactants
  IkBeps: 1.00

***v42: IKK_IkBalpha -> IKK
Excess moieties in reactants
  IkBalpha: 1.00

***v43: IKK_IkBbeta -> IKK
Excess moieties in reactants
  IkBbeta: 1.00

***v44: IKK_IkBeps -> IKK
Excess moieties in reactants
  IkBeps: 1.00
```

Fig. S5: *Full report from* `moiety_analysis` *analyzing BIOMD0000000140.*

A second case study is BIOMD0000000293 (Proctor *et al.* [2010]), which models the ubiquitin-proteasome system and its role in protein aggregation and degrading damaged proteins. Details of this case study are in the subdirectory `293` under `moiety_examples`. The model has 316 reactions (including 10 boundary reactions) and 136 chemical species. As with the previous example,

most reactions use the NC-1 convention discussed in the main text, and well over half of the reactions are moiety balanced. An example is `Monoubiquitination: E2_Ub + E3_MisP -> E3_MisP_Ub + E2`. We identified 63 candidate moieties; they can be found in the file in `moieties.yml`. Many of these are repetitive, such as `U1, U2, U3, U4, U5`. We ran `make_moiety_structures` for `moieties.yml`, but some manual effort was required to fully expose the moiety structure of all chemical species, such as the explicit representation of the moiety structures of `ATP, ADP, AMP`. We are exploring new features of `make_moiety_structure` to minimize such edits. As in the preceding example, some candidate moieties are not true chemical moieties. For example, `dam` and `damaged` indicate damaged proteins, and `Proteasome` indicates a compartment. A full list of these non-moiety candidates is: `Agg, agg, damaged, dam, misfolded, Mis, Nat, Proteasome, Seq, Sink, Source, upreg`. If we run `moiety_analysis` after incorporating these changes into the `ignored_moieties` section of `config.yml`, we get 137 reactions with mass imbalances. It turns out that for over half of the imbalanced reactions, inorganic phosphate (designated by `P` in this model) is implicit, and so we add `P` to `ignored_moieties`. Running the tool again, we find 68 imbalanced reactions, as reported in `result.txt`. We see a number of themes to the imbalanced reactions. For example,

```
UCHL1DamagedLysosomalDegradation: UCHL1_damaged + Lysosome
    -> Lysosome
```

does not consider damaged proteins transported into the lysosome. Such omissions occur in several other reactions as well. Another common cause of mass imbalance is neglecting the mass of ubiquitin ligase (`E3` in the model). This species often appears as a reactant but not as a product, as in

```
SUBInclusionGrowth1: E3SUB_SUB_misfolded + SeqAggP
    -> 2 SeqAggP + aggSUB
```

We have done approximately 10 case studies. They can be found in the `examples` folder of our `github` repository.

S5 Additional GAMES Case Studies

Our first case study is the model in Fig. 3, BIOMD000000167 (Li *et al.* [2010]). The full model has seven reactions and seven chemical species. This model has a stoichiometric inconsistency, a fact that is far from obvious. Even more surprising is the difficulty of finding the cause of the inconsistency by a manual inspection.

Fig. S6 displays the GAMES report for BIOMD000000167. There are four sections. The first reports the reactions and species in the RIS and SIS. The remaining three sections detail how these isolation sets are constructed. The second section lists the uni-uni reactions from which MEQs are constructed to form the SIS. Section three displays the MEQ substituted pseudo reactions that constitute the RIS. Finally, section four displays a linear combination of reactions in the RIS that result in a mass balance error. In this case, the stoichiometric inconsistency is detected as the creation of mass.

Our second case study is a much larger model, BIOMD000000049 (Sasagawa *et al.* [2005]). This model simulates extracellular-signal-regulated kinase (ERK) signalling networks. The model has 150 reactions and almost 100 chemical species. Here, the RIS consists of five reactions: `J44`, `J46`, `J88`, `J112`, and `J164`. The GAMES report shows that from these reactions we can infer the pseudo reaction `proteosome ->` ; that is, mass is destroyed.

To assess scaling, we ran GAMES on several models in the BiGG repository. The results are displayed in Tab. S1. iCN718 is a gemone-scasle model of Acinetobacter baumannii AYE (Norsigian *et al.* [2018]), comprised of 709 genes, 888 metabolites, and 1,105 reactions. We ran GAMES three times on this model since there was some variability in the decomposition of the stoichiometry matrix. The average runtime per iteration was 56.15 seconds. Each iteration found 1 to 2 stoichiometric inconsistencies. The average size of the RIS was approximately 76.8 reactions.

The largest model in BiGG is Recon3D (Brunk *et al.* [2018]): 10,600 reactions, 5,835 unique metabolites, and 2,248 genes. A GAMES analysis of Recon3D took 6.93 hours; several stoichiometric inconsistencies were discovered, with a mean RIS of 837.6. To put the runtime into perspective, it takes LP analysis approximately 3 hours to detect an error in Recon3D, even though LP analysis only does error detection. The computational complexity of xLP prevented us from running it on Recon3D (as detailed in Section S7.) Our future work will investigate ways to address models with large RIS and SIS.

```
We detected a mass imbalance
:  -> species_test

from the following reaction isolation set.

1. statPhosphorylation: stat_sol -> Pstat_sol + species_test
2. PstatDimerisation: 2.00 Pstat_sol -> PstatDimer_sol
3. PstatDimerisationNuc: 2.00 Pstat_nuc -> PstatDimer_nuc

----------------------------------------------------------------------
----------------------------------------------------------------------

These uni-uni reactions created mass-equivalence.
(The chemical species within a curly bracket have the same atomic mass.)

{PstatDimer_nuc=PstatDimer_sol} is inferred by:
4. PstatDimer__import: PstatDimer_sol -> PstatDimer_nuc

{Pstat_nuc=stat_nuc=stat_sol} is inferred by:
5. statDephosphorylation: Pstat_nuc -> stat_nuc
6. stat_export: stat_sol -> stat_nuc

----------------------------------------------------------------------

Based on the uni-uni reactions above, we create
mass-equivalent pseudo reactions.

(pseudo 1.) statPhosphorylation:
          {Pstat_nuc=stat_nuc=stat_sol} -> {Pstat_sol} + {species_test}
(pseudo 2.) PstatDimerisation:
                    2.00 {Pstat_sol} -> {PstatDimer_nuc=PstatDimer_sol}
(pseudo 3.) PstatDimerisationNuc:
  2.00 {Pstat_nuc=stat_nuc=stat_sol} -> {PstatDimer_nuc=PstatDimer_sol}

----------------------------------------------------------------------

An operation between the pseudo reactions:
1.00 * statPhosphorylation + 0.50 * PstatDimerisation -
0.50 * PstatDimerisationNuc

will result in empty reactant with zero mass:

:  -> {species_test}
```

Fig. S6: *Example of GAMES report when running* `SBMLLint`*. The report has four sections. The first section displays the isolation set. The remaining sections provide details of how the isolation set was constructed.*

S6 `SBMLLint`

We have created an open source implementation of moiety analysis and GAMES; these are available in the `github` repository

```
We detected a mass imbalance
:  -> M_h_c

from the following reaction isolation set.

1. R_SEAHCYSHYD: M_h2o_c + M_seahcys_c -> M_adn_c + M_selhcys_c
2. R_SEAHCYSHYD_1: M_h2o_c + M_seahcys_c -> M_adn_c + M_h_c + M_selhcys_c

------------------------------------------------------------------------
------------------------------------------------------------------------

These uni-uni reactions created mass-equivalence.
(The chemical species within a curly bracket have the same atomic mass.)

{M_h2o_c=M_h2o_e} is inferred by:
3. R_H2Ot: M_h2o_e -> M_h2o_c

------------------------------------------------------------------------

Based on the uni-uni reactions above, we create
mass-equivalent pseudo reactions.

(pseudo 1.) R_SEAHCYSHYD:
           {M_h2o_c=M_h2o_e} + {M_seahcys_c} -> {M_selhcys_c} + {M_adn_c}
(pseudo 2.) R_SEAHCYSHYD_1:
 {M_h2o_c=M_h2o_e} + {M_seahcys_c} -> {M_selhcys_c} + {M_adn_c} + {M_h_c}

------------------------------------------------------------------------

An operation between the pseudo reactions:
-1.00 * R_SEAHCYSHYD + 1.00 * R_SEAHCYSHYD_1

will result in empty reactant with zero mass:

:  -> {M_h_c}
```

Fig. S7: *GAMES report for BiGG model* `iCN718`*. The report has four sections: (1) the summary (at the top); (2) the uni-uni reactions used to construct MEQs (below the double lines); (3) MEQ substituted pseudo reactions; and (4) a linear combination of the reactions in (3) that violate the GAMES detect criteria by creating or destroying mass.*

`https://github.com/ModelEngineering/SBMLLint`. The term "lint" in the name comes from software engineering; it refers to tools that do static error checking. Unlike systems such as `MEMOTE` that do static error checking as part of a bigger system, `SBMLLint` operates as a stand-alone package that can be used in isolation. `SBMLLint` contains several command line tools. The tools can be installed using the command line `pip install SBMLLint`.

Model	genes	metabolites	reactions	GAMES time	num.errors	RIS
iCN718	709	888	1015	56.15 sec	1.3	76.8
iECIAI1_1343	1343	1968	2765	891.50 sec	213.0	555.3
iCHOv1	1766	4456	6663	2.94 hr	235.7	468.6
Recon3D	2248	5835	10600	6.93 hr	528	837.6

Table S1: Evaluation of GAMES on four BiGG models. All results were from xGAMES, as bGAMES did not find errors in the above models. Runtime and RIS size were calculated by averaging three runs for each model. Recon3D was only run once.

- `moiety_analysis` takes as input an SBML file and optionally a configuration file (which may contain explicit representations of moiety structures of chemical species); it outputs a report of moiety balance errors.
- `games` takes as input an SBML file and optionally a configuration file; it outputs a report of stoichiometric inconsistencies.
- `lp_analysis` takes as input an SBML file; it outputs a report of stoichiometric inconsistencies detected.
- `print_reactions` takes as input an SBML file; it outputs formatted text for the reactions in the model.
- `make_moiety_structure` takes as input an SBML file and a YAML file listing moieties; it outputs the `moiety_structure` section of the configuration file.

`SBMLLint` also provides a python API to faciliate use in Jupyter notebooks (Pérez and Granger [2007]). In addition to XML files, all tools can instead take as input the user-friendly antimony modeling language (Smith *et al.* [2009]). Further details can be found in the `github` repository.

S7 Isolating Errors With xLP analysis

We use the term **LP analysis** to refer to the algorithm that detects stoichiometric inconsistencies using linear programming (LP). The algorithm runs an LP to check feasibility for the following optimization:

$$\begin{array}{ll} \text{Minimize} & \sum_{i=1}^{m} m_i \\ \text{Subject to} & N^{\mathrm{T}} m = 0 \\ \text{Where} & m_i \geq 1 : 1 \leq i \leq m. \end{array} \tag{1}$$

There are extensions to LP analysis that have been proposed for isolating stoichiometric inconsistencies. The **MILP (mixed integer linear program-**

ming) extended LP analysis (xLP) requires doing three MILP optimizations beyond the LP feasibility analysis above (Gevorgyan *et al.* [2008]):

- Step 1: Find unconserved metabolites for all stoichiometric inconsistencies in the model. That is, find the union of the species isolation sets (SIS) for stoichiometric inconsistencies in the model.
- Step 2: Detect inconsistent minimal net stoichiometries. This finds the SIS for each stoichiometric inconsistency.
- Step 3: Find elementary leakage modes. This calculates the reaction isolation set (RIS) for each stoichiometric inconsistency.

Below, we replicate the optimization problems formulated in Gevorgyan *et al.* [2008], along with some explanations of the variables used. Step 1 solves the following optimization:

$$\begin{array}{ll} \text{Maximize} & \sum_{i=1}^{m} k_i \\ \text{Subject to} & N^{\mathrm{T}} m = 0 \\ \text{Where} & 0 \leq k_i \leq m_i, k_i \in \{0,1\}; 1 \leq i \leq m \end{array} \quad (2)$$

where: i indexes chemical species; m is a vector of chemical species masses; and m_i is the mass of species i in m. Although somewhat confusing, m is also used to indicate the length of the vector m. Step 2 operates on K, the left nullspace matrix of the stoichiometry matrix N:

$$\begin{array}{ll} \text{Minimize} & \sum_{i=1}^{m} k_i \\ \text{Subject to} & y^{\mathrm{T}} K = 0, y_j \geq \epsilon \\ \text{Where} & 0 \leq y_i \leq k_i, k_i \in \{0,1\}; 1 \leq i \leq m. \end{array} \quad (3)$$

where: ϵ represents a small positive number; i is the same as in Step 1; and j indexes unconserved metabolites. Step 3 iterates across all minimal net stoichiometry y, solving an MILP for each to find the flux rate $v_i > 0$:

$$\begin{array}{ll} \text{Minimize} & \sum_{i=1}^{m} k_i \\ \text{Subject to} & (N|y)v = 0, v_j \geq \epsilon \\ \text{Where} & 0 \leq v_i \leq k_i, k_i \in \{0,1\}; 1 \leq i \leq m. \end{array} \quad (4)$$

There are a number of significant shortcomings with xLP analysis. First, for biological feasibility, detecting inconsistent minimal net stoichiometries (Step 2) should assign to each metabolite a non-negative value (the m_i). However, the calculation of the left nullspace matrix does not ensure that the values are non-negative.

A second concern is computational complexity. Step 3 requires doing an MILP *for each* inconsistent minimal net stoichiometry (the result of Step 2).

This can lead to extremely long runtimes. For example, the Recon3D model in BiGG has 4,357 unconserved metabolites, and we found that a single MILP iteration on a modern laptop takes about 145 seconds. This implies a total runtime of over 7 days! In contrast, GAMES analyzes this model in a little less than 7 hours.

Another concern with xLP analysis is that it does not provide an explanation for how the stoichiometric inconsistencies relate to chemical species (SIS) and reactions (RIS). This means that modelers have no guidance as to alternative approaches for remediating errors, as is provided by the narrative in Fig. 2. Even worse, modelers cannot validate that an error truly exists, which is a concern because of possible numerical issues in matrix operations that can result in false positives.

Last, although xLP was proposed over a decade ago, we are unaware of any system that implements it. MEMOTE implements the first step in xLP (finding unconserved metabolites), but the notes in their code suggest that this step is not functional in the MEMOTE system. (See `https://memote.readthedocs.io /en/latest/_modules/memote /support/consistency.html #check_stoichiometric_consistency`, which, as of July 8, 2020, has a 'FIXME' flag.) A more complete implementation is described in documentation for the COBRA Open Toolbox. (See `https://opencobra.github.io/cobratoolbox /stable/modules /reconstruction/modelGeneration /stoichConsistency/index.html`.) This implementation does not use Step 3 as described in Gevorgyan *et al.* [2008] to find error causing reactions (RIS), and so their version of Step 3 avoids much of the computational complexity described above. We note that even with this revised Step 3, the algorithm does not provide a narrative that explains errors in terms of the SIS and RIS. Further, we are unaware of any publication that describes and analyzes this version of Step 3 in xLP.

S8 BioModels Statistics

Our studies use the curated SBML models in BioModels. We focus on curated models because of the human effort expended to validate their correct operation. These models address a wide range of biological processes, including metabolism, signaling, and motility. We have applied LP analysis to the curated models, and found that approximate 20% of the models have at least one stoichiometric inconsistency. This does not necessarily mean that the models are in error in terms of the studies for which they were designed. However, it does raise concerns for researchers considering reuse of these models for new studies.

Tab. S2 displays statistics for the types of reactions of these models. We note that there is considerable variability in the number of reactions of each type. Even so, we see that multi-multi reactions are relatively uncommon, accounting for about 8% of all models. If more multi-multi reactions were present, it is likely that bGAMES would have lower coverage since bGAMES does not consider multi-multi reactions.

Measure	Uni-Uni	Multi-Uni	Multi-Multi	Boundary	Total
Count	10.6	11.6	4.0	6.4	32.6
Fraction	0.33±0.32	0.22±0.29	0.079±0.18	0.38±0.40	1.000

Table S2: *Average number and average fraction (± standard deviation) of reactions by type in the 826 curated models from BioModels. Count is the average number of each reaction type, and Fraction is the average fraction of each reaction type across models.*

Fig. S8 displays a bar plot of the fractions of reactions of each type. We see that uni-uni and multi-uni reactions account for the majority of the reactions in curated BioModels. This explains why bGAMES has a coverage as high as 77.8% in BioModels.

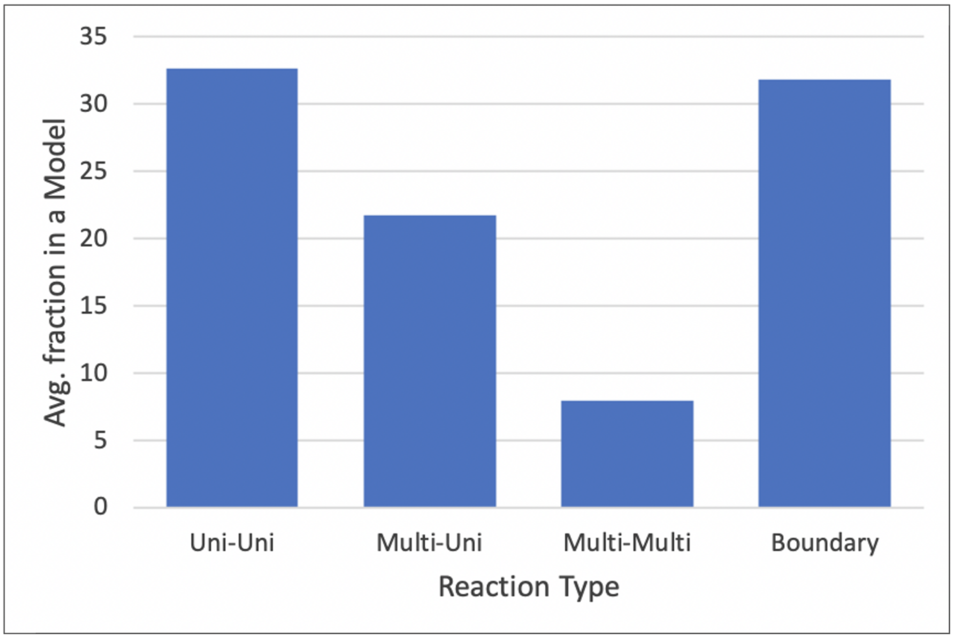

Fig. S8: *Bar plot of the fraction of reactions of the different types for a model in BioModels.*

S9 Future Work

This section provides more details thoughts on our future work.

1. For large models, such as those in BiGG, there may be a way to integrate GAMES with mathematical programming or other methods. For example,

GAMES creates MEQs of chemical species using uni-uni reactions, reducing the total number of reactions that need to be examined. Applying LP on this reduced model - a model comprised only of multi-uni reactions and multi-multi reactions of MEQs - may benefit from the reduced dimension. We are seeking for fundamental ways to improve both the accuracy and the speed of the GAMES algorithms.

2. Another consideration for large models is the very large size of the RIS and SIS, often occurring in the genome-scale metabolic models. Many such models are, in part, generated automatically. We speculate that one reason for large isolation sets may be related to the manner in which automatic generation of models is done. Deeper understanding of such systematic characteristics would help us improve current error isolation algorithms and suggest more effective error remediation for modelers.

References

Brunk, E., Sahoo, S., Zielinski, D. C., Altunkaya, A., Dräger, A., Mih, N., Gatto, F., Nilsson, A., Preciat Gonzalez, G. A., Aurich, M. K., Prlić, A., Sastry, A., Danielsdottir, A. D., Heinken, A., Noronha, A., Rose, P. W., Burley, S. K., Fleming, R. M. T., Nielsen, J., Thiele, I., and Palsson, B. O. (2018). Recon3D enables a three-dimensional view of gene variation in human metabolism. *Nature Biotechnology*, **36**(3), 272–281.

Gevorgyan, A., Poolman, M. G., and Fell, D. A. (2008). Detection of stoichiometric inconsistencies in biomolecular models. *Bioinformatics*, **24**(19), 2245–2251.

Hoffmann, A., Levchenko, A., and Scott, M. L. (2007). The I K B - N F - K B Signaling Module : Temporal Control and Selective Gene Activation. **298**(6), 1241–1246.

Li, C., Donizelli, M., Rodriguez, N., Dharuri, H., Endler, L., Chelliah, V., Li, L., He, E., Henry, A., Stefan, M. I., Snoep, J. L., Hucka, M., Le Novère, N., and Laibe, C. (2010). BioModels Database: An enhanced, curated and annotated resource for published quantitative kinetic models. *BMC systems biology*, **4**, 92.

Norsigian, C. J., Kavvas, E., Seif, Y., Palsson, B. O., and Monk, J. M. (2018). icn718, an updated and improved genome-scale metabolic network reconstruction of acinetobacter baumannii aye. *Frontiers in Genetics*, **9**, 121.

Pérez, F. and Granger, B. E. (2007). IPython : A System for. *IEEE Journals & Magazines*, **9**(3), 21–29.

Proctor, C. J., Tangeman, P. J., and Ardley, H. C. (2010). Modelling the role of UCH-L1 on protein aggregation in age-related neurodegeneration. *PLoS ONE*, **5**(10).

Sasagawa, S., Ozaki, Y.-i., Fujita, K., and Kuroda, S. (2005). Prediction and validation of the distinct dynamics of transient and sustained ERK activation. *Nature Cell Biology*, **7**(4), 365–373.

Smith, L. P., Bergmann, F. T., Chandran, D., and Sauro, H. M. (2009). Antimony: a modular model definition language. *Bioinformatics (Oxford, England)*, **25**(18), 2452–2454.
